# Memory: An Extended Definition

**DOI:** 10.3389/fpsyg.2019.02523

**Published:** 2019-11-07

**Authors:** Gregorio Zlotnik, Aaron Vansintjan

**Affiliations:** ^1^Clinique de la Migraine de Montreal, Montreal, QC, Canada; ^2^Department of Film, Media and Cultural Studies, Birkbeck, University of London, London, United Kingdom

**Keywords:** memory, extended cognition, incorporation, storage of information, information technology, experience

## Abstract

Recent developments in science and technology point to the need to unify, and extend, the definition of memory. On the one hand, molecular neurobiology has shown that memory is largely a neuro-chemical process, which includes conditioning and any form of stored experience. On the other hand, information technology has led many to claim that cognition is also extended, that is, memory may be stored outside of the brain. In this paper, we review these advances and describe an extended definition of memory. This definition is largely accepted in neuroscience but not explicitly stated. In the extended definition, memory is the capacity to store and retrieve information. Does this new definition of memory mean that *everything* is now a form of memory? We stress that memory still requires incorporation, that is, *in corpore*. It is a relationship – where one biological or chemical process is incorporated into another, and changes both in a permanent way. Looking at natural and biological processes of incorporation can help us think of how incorporation of internal and external memory occurs in cognition. We further argue that, if we accept that there is such a thing as the storage of information outside the brain – and that this organic, dynamic process can also be called “memory” – then we open the door to a very different world. The mind is not static. The brain, and the memory it uses, is a work in progress; we are not now who we were then.

## Introduction

In the short story “Funes, the memorious,” Jorge Luis Borges invites us to imagine a man, Funes, who cannot forget anything. The narrator is ashamed in the inexactness of his retelling: his own memory is “remote and weak,” in comparison to that of his subject, which resembles “a stammering greatness.” Unlike Funes, he says, “we all live by leaving behind” – life is impossible without forgetting. He goes on to note that, even though Funes could remember every split second, he couldn’t classify or abstract from his memories. “To think is to forget a difference, to generalize, to abstract.” The reader may be led to wonder how Funes’ brain has the capacity to store all of that memory. doesn’t it reach its limits at some point? Borges leaves that question to our imagination.

In popular culture, memory is often thought of as some kind of physical thing that is stored in the brain; a subjective, personal experience that we can recall at will. This way of thinking about memory has led many to wonder if there is a maximum amount of memories we can have. But, this idea of memory is at odds with advances in the science of memory over the last century: memory isn’t really a fixed thing stored in the brain, but is more of a chemical process between neurons, which is not static. What’s more, advances in information technology are pushing our understanding of memory into new directions. We now talk about memory on a hard drive, or as a chemical change between neurons. Yet, these different definitions of memory continue to co-exist. A more narrow definition of memory, as the storage of experiences in the brain, is increasingly at odds with an extended definition, which acknowledges these advances. However, while this expanded definition is often implicitly used, it is rarely explicitly acknowledged or stated. Today, the question is no longer, how many memories can we possibly have, but, how is the vast amount of memory we process on a daily basis integrated into cognition?

In this paper, we outline these advances and the currently accepted definitions of memory, arguing that these necessarily imply that we should today adopt an extended definition. In the following, we first describe some key advances in the science of memory, cognitive theory, and information technology. These suggest to us that we are already using a unified, and extended, definition of memory, but rarely made explicit. Does this new definition of memory mean that *everything* is now a form of memory? We argue that looking at natural and biological processes of incorporation can help us think of how incorporation of internal and external memory occurs in cognition. Finally, we note some of the implications of this extended definition of memory.

## Background: Advances in the Science of Memory

Already in the 19th century, the recognition that the number of neurons in the brain doesn’t increase significantly after reaching adulthood suggested to early neuroanatomists that memories aren’t primarily stored through the creation of neurons, but rather through the strengthening of connections *between* neurons (Ramón y Cajal, 1894). In 1966, the breakthrough discovery of long-term potentiation (LTP) suggested that memories may be encoded in the strength of synaptic signals between neurons (Bliss and Lømo, 1973). And so we started understanding memory as a neuro-chemical process. The studies by Eric Kandel of the *Aplysia californica*, for which he won the Nobel prize, for example, show that classical conditioning is a basic form of memory storage and is observable on a molecular level within simple organisms (Kandel et al., 2012). This in effect expanded the definition of memory to include storage of information in the neural networks of simple lifeforms. Increasingly, researchers are exploring the chemistry behind memory development and recall, suggesting these molecular processes can lead to psychological adaptations (e.g., Coderre et al., 2003; Laferrière et al., 2011).

Memory is today defined in psychology as the faculty of encoding, storing, and retrieving information (Squire, 2009). Psychologists have found that memory includes three important categories: sensory, short-term, and long-term. Each of these kinds of memory have different attributes, for example, sensory memory is not consciously controlled, short-term memory can only hold limited information, and long-term memory can store an indefinite amount of information.

Key to the emerging science of memory is the question of how memory is consolidated and processed. Long-term storage of memories happens on a synaptic level in most organisms (Bramham and Messaoudi, 2005), but, in complex organisms like ourselves, there is also a second form of memory consolidation: *systems consolidation* moves, processes, and more permanently stores memories (Frankland and Bontempi, 2005). Today, there are many models of how memory is consolidated in cognition. Single-system models posit that the hippocampus supports the neocortex in encoding and storing long-term memories through strengthening connections, finally leading the memory to become independent from the hippocampus (Ibid.). Multiple-trace theory instead proposes that each memory has a unique code or memory trace, which continues to involve the hippocampus to an extent (Hintzman and Block, 1971; Hintzman, 1986, 1990; Whittlesea, 1987; Versace et al., 2014; Briglia et al., 2018). In another theory, memory is understood as a form of negative entropy or rich energy (Wiener, 1961, 1988), which is then processed in a way that minimizes the expenditure of energy by the brain (Friston, 2010; Van der Helm, 2016). Our heightened capacity to store information may be due to our ability to reduce disorder and process large amounts of information rapidly, a necessarily non-linear process (Wiener, 1961, 1988). The forgetting and fading of memories is also understood as being an important aspect of the functioning and utility of these memories (Staniloiu and Markowitsch, 2012). As with a computer hard drive, memories can also be “corrupted” – false memories are commonly studied within forensic psychology (Loftus, 2005). Together, these advances highlight how different kinds of memory storage are non-linear – that is, subject to complex systems interactions – contextual, and plastic. They also shed light on why, and how, we are able to live with such large quantities of information. It may not be that Funes has the special ability to remember everything, but that he *lacks* our ability to incorporate, and sort through, a potentially infinite amount of information.

The advance of the fields of genetics and epigenetics has also given us new metaphors to describe memory. We understand DNA as a structure that carries information that we call “genetic code” – kind of like a computer chip for biological processes. Today, the metaphor has come full circle and we can now use DNA to store and extract digital data (Church et al., 2012). The study of epigenetics suggests that simple lifeforms pass on memories across generations through genetic code (Klosin et al., 2017; Posner et al., 2019), suggesting a need to study whether humans and other complex life forms may do so as well. With these advances, our understanding of how memory is stored has expanded once again.

Further, we can now store memory in places that we haven’t been able to before. Smartphones, mind-controlled prosthetic limbs, and Google Glasses all offer new ways to store information and thereby interact with our surroundings. Our ability to produce information alters how we perceive the world, with far-reaching implications. As Stephen Hawking, the Nobel prize-winning physicist explained in his 1996 lecture, “Life in the universe,”

What distinguishes us from [our ancestors], is the knowledge that we have accumulated over the last 10000 years, and particularly, over the last three hundred. I think it is legitimate to take a broader view, and include externally transmitted information, as well as DNA, in the evolution of the human race (Hawking, 1996).

The sheer quantity of available information today, as well as developments in an understanding of memory – from fixed and physical to dynamic, chemical, and a process of rich energy transfer – lead to a very different picture of memory than the one we had 100 years ago. Memory seems to exist everywhere, from an *Aplysia*’s ganglion to DNA to a hard drive.

To account for these developments, cognitive scientists now propose that human cognition is actually extended beyond the brain in ways that theories of the mind did not previously recognize (Clark and Chalmers, 1998; Clark, 2008). This approach is being called 4E cognition (Embodied, Embedded, Extended, and Enactive). For example, enactivism posits that cognition is a dynamic interaction between an organism and its environment (Varela et al., 1991; Chemero, 2009; Menary, 2010; Rowlands, 2010; Favela and Chemero, 2016; Briglia et al., 2018). According to this framework, cognition is a process of incorporation between the environment and the body/brain/mind. To be clear, cognition is not incorporated *in* the surroundings, only the *corpus* can incorporate, and thus cognition (or what we call “mind”) is a product of the interaction between the brain, the body, and the environment.

## Extending Memory

These developments indicate that we need to reconceptualize our definition of memory. What is the difference between trying to recall a childhood experience, and searching for an important email archived years ago? This distinction is best represented through the difference in how we use the words “memory” and “memories.” Usually, “memories” tends to refer to events recalled from the past, which are seen as more representational and subjective. In contrast, “memory” now is used to refer to storage of information *in general*, including in DNA, digital information storage, and neuro-chemical processes. Today, science has moved far beyond a popular understanding of memory as fixed, subjective, and personal. In the extended definition, it is simply *the capacity to store and retrieve information*. To illustrate why memory has extended beyond this original use, we want to ask the reader: what do a stressed-out driver and a snail have in common?

(1). A homeowner has been trying to sell her house for a year, and worrying about it. One day, she’s driving to work and becomes extremely anxious, for no apparent reason. She wasn’t thinking of anything in particular at the time. Confused, she looks around, and notices a billboard advertising a real estate agency. She realizes that she had seen it out of the corner of her eye, and her brain had then processed the information while she was thinking of something else, which then triggered the anxiety attack.

(2). Consider a nerve cell of an *A. californica*, a kind of sea snail, which is prodded vigorously for a short time period, provoking an immediate withdrawal response. Shortly afterward, it is prodded less intensely, but, it elicits the same withdrawal response. It is found that the slugs’ nerve cell is sensitive for up to 24 h – the nerve cells “remember” past pain.

Each example illustrates a different kind of chemical, biological process. In the first example, an outside stimulus triggers a stress response for the homeowner. We can surmise that though she didn’t “remember” anything, non-consciously, she did. In the second example, the snail certainly “remembers” the provocation, even though this memory is only stored in a few cells. But can we really call this memory?

However, on closer examination, we are forced to concede that each of them should be called a form of memory. First, consider the homeowner: her brain “remembers” something that does not occur to her as a conscious thought. It is clearly a chemical process occurring in the background. Most would grant that this would nevertheless be a form of memory, as it involves recalling information stored in her brain. Already, a broader definition of memory is used that does not imply conscious attention. Now, consider the snail: it is also storing information chemically. Once again, this does not involve a conscious, subjective process of storing and remembering – it is purely reactive, but information is being stored and recalled nonetheless. We would need to concede that if the homeowner’s experience counts as memory, then the slug’s automatic response does as well. There is in fact little difference between the first two examples: there is a transfer of information that causes a reaction. Both should be considered forms of memory.

## A Slippery Slope?

If we agree with this expanded definition of memory, then it follows that *experience* is also a form of stored information, kinds of *memory*. We are not saying that a particular experience, as an *event*, is a memory. Rather, we here use the word “experience” as connoted by the phrase “an experienced driver,” an “experienced writer.” They have a set of experiences, remembered through practice, and retrieved when they drive, or write. When we accumulate knowledge, information, and techniques, then the accumulation of those separate processes constitute *experience*. This experience involves retrieval of information, conversely, being experienced is the process of retrieving memory.

Under this definition, even immunological and allergy processes may be considered memory. There is a storage of information of the allergen or the viral/bacterial aggressor and when the aggressor or allergen re-appears there is a cascade of inflammatory processes. This can be considered the storage and retrieval of information, and thus a form of memory. This does not contradict the accepted definition of memory within psychology, as it is still seen as the ability to encode, store, and recall information. Rather, it extends it to processes not just bound by the brain.

If memory is indeed defined as “the capacity to store and/or retrieve information,” then this may lead anyone to ask – what *isn’t* memory? Wouldn’t this definition of memory be far too broad, and include a vast range of phenomena? Is the extended definition of memory, as is being proposed by neurobiologists and cognitive theorists, a slippery slope?

As we suggested above, however, memory still involves a process of incorporation, that is, requiring a *corpus*. While memory may be stored on the cloud, it requires a system of incorporation with the body and therefore the mind. In other words, the “cloud” by itself is not memory, but operates through an infrastructure (laptops, smart phones, Google Glasses) that are integrated with the brain-mind through learned processes of storage and recall. The conditioning of an *Aplysia*’s ganglion is incorporated into an organism. Memory, it seems, is not just mechanistic, but a dynamic process. It is a relationship – where one biological or chemical process is incorporated into another, and changes both in a permanent way. A broadened definition must account for this dynamic relationship between organisms and their environment.

How can we understand this process of incorporation? It appears that symbiotic incorporation of biological processes is quite common in nature. Recent studies offer more evidence that early cells acquired mitochondria by, at some point, incorporating external organisms into their own cell structure (Thrash et al., 2011; Ferla et al., 2013). Mitochondria have their own genome, which is similar to that of bacteria. What was once a competitor and possibly a parasite became absorbed into the organism – and yet, the mitochondrion was not fully incorporated and retains many of its own processes of self-organization and memory storage, separate from the cell it resides in. This evolutionary process highlights the way by which external properties may become incorporated into the internal, changing both. Looking at natural and biological processes of incorporation can help us think of how incorporation of internal and external memory occurs in cognition.

## Implications

This extended definition of memory may seem ludicrous and hard to accept. You may be tempted to throw up your hands and go back to the old, restricted, definition of memory – one that requires the transmission of subjective memories.

We beg you not to. There are several benefits of this approach to memory. First, in biology, expanding the definition of memory helps us shift from a focus on “experience” (which suggests an immaterial event) to a more material phenomenon: a deposit of events that may be stored and used afterward. By expanding the concept of memory, the study of memory within molecular neurobiology becomes more relevant and important. This expanded definition is in large part already widely accepted, for example, in Kandel’s *Aplysia*, conditioning is acknowledged to be a part of memory, and memory is not a part of conditioning. Memory would become the umbrella for learning, conditioning, and other processes of the mind/brain. Doing so changes the frame of observation from one which understands memory as a narrow, particular process, to one which understands it as a dynamic, fluid, and interactive phenomenon, neither just chemical or digital but integrated into our experience through multiple media. Second, it helps to conceptualize the relationship between biology, psychology, cognitive science, and computer science – as all three involve studying the transfer of information.

Third, it opens up an interesting way to imagine our own future. If we accept that there is such a thing as the storage of information outside the brain – and that this organic, dynamic process can also be called “memory” – then we open the door to a very different world. The mind is not static. Rather, like early cells acquiring mitochondria, it incorporates information from its surroundings, which in turn changes it. The brain, and the memory it uses, is a work in progress; we are not now who we were then. Many have already noted the extent to which we are cyborgs (Harraway, 1991; Clark, 2003, 2005); this neat line between human and technology may become more and more blurred as we develop specialized tools to store all kinds of information in our built environment. In what ways will the mind-brain function differently as it becomes increasingly more incorporated in its milieu, relying on it for information storage and processing?

Now let’s talk about Funes. His inability to forget his memories may seem familiar to some, a metaphor for our current condition. We may now recognize a bit of ourselves in him: we don’t see limits in our capacity to store new information, and the sheer availability of it is sometimes overwhelming. Even without the arrival of the Information Age, we carry with us through life a heavy load of disappointments, broken dreams, little tragedies and many memories. We know that forgetting is a must and a challenge. Yet, we are learning rapidly how to incorporate and use the massive amounts of data now available to us. The main challenge for each of us is to harness and control the unleashed powers given to us by technology. The future is uncertain, but some things remain the same. As Kandel (2007, p. 10) wrote, “We are who we are in great measure because of what we learn, and what we remember.”

## Author Contributions

GZ and AV drafted and edited the manuscript. Both authors contributed to manuscript revision, read, and approved the submitted version.

## Conflict of Interest

The authors declare that the research was conducted in the absence of any commercial or financial relationships that could be construed as a potential conflict of interest.
